# Identification of selective sweeps reveals divergent selection between Chinese Holstein and Simmental cattle populations

**DOI:** 10.1186/s12711-016-0254-5

**Published:** 2016-10-06

**Authors:** Minhui Chen, Dunfei Pan, Hongyan Ren, Jinluan Fu, Junya Li, Guosheng Su, Aiguo Wang, Li Jiang, Qin Zhang, Jian-Feng Liu

**Affiliations:** 1 Department of Animal Genetics, Breeding and Reproduction, China Agricultural University, Beijing, 100193 China; 2 Center for Quantitative Genetics and Genomics, Department of Molecular Biology and Genetics, Aarhus University, AU-Foulum, 8830 Tjele, Denmark; 3 National Natural Science Foundation of China, Beijing, 100085 China; 4 Institute of Animal Science, Chinese Academy of Agricultural Science, Beijing, 100193 China

## Abstract

**Background:**

The identification of signals left by recent positive selection provides a feasible approach for targeting genomic variants that underlie complex traits and fitness. A better understanding of the selection mechanisms that occurred during the evolution of species can also be gained. In this study, we simultaneously detected the genome-wide footprints of recent positive selection that occurred within and between Chinese Holstein and Simmental populations, which have been subjected to artificial selection for distinct purposes. We conducted analyses using various complementary approaches, including LRH, XP-EHH and F_ST_, based on the Illumina 770K high-density single nucleotide polymorphism (SNP) array, to enable more comprehensive detection.

**Results:**

We successfully constructed profiles of selective signals in both cattle populations. To further annotate these regions, we identified a set of novel functional genes related to growth, reproduction, immune response and milk production. There were no overlapping candidate windows between the two breeds. Finally, we investigated the distribution of SNPs that had low F_ST_ values across five distinct functional regions in the genome. In the low-minor allele frequency bin, we found a higher proportion of low-F_ST_ SNPs in the exons of the bovine genome, which indicates strong purifying selection of the exons.

**Conclusions:**

The selection signatures identified in these two populations demonstrated positive selection pressure on a set of important genes with potential functions that are involved in many biological processes. We also demonstrated that in the bovine genome, exons were under strong purifying selection. Our findings provide insight into the mechanisms of artificial selection and will facilitate follow-up functional studies of potential candidate genes that are related to various economically important traits in cattle.

**Electronic supplementary material:**

The online version of this article (doi:10.1186/s12711-016-0254-5) contains supplementary material, which is available to authorized users.

## Background

The patterns of genetic variation are essential for understanding the history and structure of populations and the relationship between genotype and phenotype [1–3]. To date, many studies have focused on the genome-wide scanning of signals that were left by recent positive selection in many species, such as humans [4–7], plants [8], and domestic animals [9, 10].

Signatures of selection in a genome usually involve three typical genomic features, i.e., high-frequency derived alleles, long-range haplotypes with strong linkage disequilibrium (LD) and highly differentiated allele frequencies between populations [6]. Specifically, a selective sweep rapidly increases the frequency of the favorable causal variant, and strong LD persists between the causal variant and neighboring polymorphisms relative to neutral regions, which results in an unusually long-range haplotype with a high level of homozygosity [4, 5]. When geographically variable selective forces or directional selection with some economic purpose favor different variants in different regions, allele frequencies in such regions will differ greatly among populations [9].

To detect these genomic features that result from recent positive selection, various analytical methods have been proposed and successfully applied to many species. These approaches are largely considered as belonging to two different types. One type is based on LD patterns across genomes, such as the long-range haplotype (LRH) test [7], integrated haplotype homozygosity score (iHS) [5], cross population extended haplotype homozygosity (XP-EHH) test [4] and Rsb test [11]. The second type is based on allele frequency, such as F_ST_ [12] and heterozygosity [13]. Among these detection approaches, the XP-EHH and Rsb tests are more sensitive to a selective sweep in which the corresponding allele has approached or achieved fixation within one population [4, 11], whereas the LRH and iHS tests have advantages in exploring selective sweeps with variants at moderate frequencies [4]. Differentiation-based methods are more powerful for detecting complex events, such as selection on standing variation [14]. Hence, a composite method combining different detection approaches to provide complementary information was considered as an optimal strategy in searching for selection signatures with different features [6].

Identification of selective signatures in the bovine genome can provide information about biologically meaningful variants that underlie adaptation to specific environments as well as human-mediated selection [3]. The bovine genome has developed extensive LD due to domestication, breed formation and the implementation of breeding programs [15, 16]. Moreover, with the advent of high-throughput genotyping technologies such as the high-density single nucleotide polymorphism (SNP) BeadChip and next-generation sequencing, in addition to the well-established analytical methods, it is becoming feasible to explore the signals of selection present in the genomes of bovine populations. Recently, many studies have been conducted to explore signatures undergoing positive selection in cattle using different methods [9, 10, 17–20].

Since the domestication of cattle 8000 to 10,000 years ago [21], strong selection through domestication and subsequent artificial selection have resulted in much diversity among present-day cattle. Diversity includes variation in morphology, physiology, production and fertility traits. To characterize the response in genetic variation to domestication and strong artificial selection, we first conducted the detection of selective signatures both within and between populations using two popular Chinese cattle breeds of European origin, i.e., Holstein and Simmental, which are typical dairy and beef/milk dual-purpose breeds with markedly different selection directions. Furthermore, the detection of selection signatures can act as a complement to current gene mapping approaches, e.g. genome-wide association studies (GWAS). The detection of selection signatures and GWAS can provide complementary information for unraveling the genetic structure of complex traits. By comparing candidate regions found through the identification of selection signatures and GWAS, we can also test the contributions of genes under selection to the phenotype, which can subsequently be used in genomic selection.

In this study, we used the 770K BovineHD BeadChip instead of the most commonly used 54K SNP genotyping panel to enable detection of selection signatures with higher accuracies. Furthermore, we adopted a composite strategy instead of a single detection method to explore potential selective footprints left within and between populations. This approach can avoid the limitations of each individual approach and achieve power gain in the detection of selection signatures. Specifically, we first conducted an LRH test to pinpoint alleles that carry unusually long haplotypes and then to identify selection signals within each breed. Subsequently, both XP-EHH and F_ST_ statistics were calculated to examine the divergent genetic variation affected by the strong directional selection between these two populations. Although the detection of a selection sweep near fixation by XP-EHH has little value for the population itself, it can still facilitate marker-assisted selection for other dairy cattle breeds and hybrids, in which the causal gene has not been selected or is still under selection. Our study aimed at identifying novel selection signals in two popular Chinese cattle populations. Our results will complement existing findings and facilitate follow-up functional genomics studies as well as marker-assisted selection in cattle breeding.

## Methods

### Animal resources and control of data quality

Data were obtained from 96 Holstein cattle, including 10 cows and 86 bulls, and 447 Simmental bulls. Genotyping was performed using the Illumina BovineHD BeadChip that includes 777, 962 SNPs. To ensure the high quality of the SNP data, a series of quality control measures was performed. The following criteria were applied for quality control: (1) an individual sample was removed when the missing genotype rate per individual was higher than 0.05; SNPs were removed (2) when the minor allele frequency (MAF) was lower than 0.05, (3) when there was no known autosomal genomic location (UMD 3.1), (4) when the SNP genotypes were not in Hardy–Weinberg equilibrium (*P* > 10^−6^). However, for the XP-EHH and F_ST_ tests, loci with a MAF lower than 0.05 were also included. Beagle (version 3.3.2) [22] was used to impute missing genotypes and infer the haplotype phase. To ensure independence among the individuals collected in both populations, a relatedness test was performed using PLINK [23]. A set of approximately independent SNPs was extracted using the PLINK option: indep-pairwise 50 5 0.2, i.e., removal of one SNP of a pair of SNPs that have a pairwise r^2^ higher than 0.2 within a window of 50 SNPs, and shifting the window by steps of five SNPs. The pairwise IBD was estimated for pairs of individuals within each population. Individuals of a pair of individuals that had a *pi*-*hat* value greater than 0.2 were considered to be closely related, and thus, one individual was removed from the analysis.

### Population structure

To characterize the origins of the Chinese Holstein and Simmental populations, we conducted a principal component analysis (PCA) using EIGENSOFT 6.0.1 [24] and breed assignment analyses using WIDDE [25]. For each population, 15 individuals were randomly selected to conduct analyses. In breed assignment analyses, the allele sharing distance (ASD) was calculated between test individuals and individuals in the world reference dataset included in WIDDE. For each test individual, the average ASD with all the individuals of each reference population was calculated and the top five genetically closest populations were summarized. As only populations with at least 15 individuals are included in the world reference dataset in WIDDE, the Simmental reference population, which included 10 individuals genotyped on the Illumina BovineHD BeadChip, although present in WIDDE was not included in the world reference dataset. Therefore we downloaded the world reference dataset and the Simmental reference population from WIDDE, and conducted PCA using EIGENSOFT rather than WIDDE. The downloaded dataset included 2513 individuals genotyped by either the Illumina BovineHD BeadChip or Illumina BovineSNP50 BeadChip. Before conducting PCA, the data was filtered. To merge our dataset and the dataset downloaded from WIDDE, we retained only common SNPs. Then, PLINK [23] was used to exclude individuals with a missing genotype rate higher than 0.05, SNPs with a missing genotype rate higher than 0.25, and SNPs with a MAF lower than 0.01.

### Haplotype-block partitioning

For the purpose of this study, we used the algorithm suggested by Gabriel et al. [26], which defines a pair of SNPs to be in ‘strong LD’ if the one-sided upper 95 % confidence bound on D’ is higher than 0.98, and the lower bound is higher than 0.7. A haplotype block is defined as a region across which 95 % of the informative SNP pairs show strong LD. LD statistics were estimated, and haplotype blocks were constructed using HAPLOVIEW [27] v4.2 based on the reconstructed haplotypes.

### Detection of selection signatures within populations

The LRH test statistics were calculated in both directions and for all SNPs using Sweep 1.1 [4], for the Holstein and Simmental populations, separately. The extended haplotype homozygosity (EHH) for each core SNP was calculated at a marker with all EHH values of 0.04, which is roughly equivalent to a genetic distance of 0.25 cM [4]. To correct for local variation in recombination rates, the EHH of the tested core allele was compared with the EHH of all other core alleles combined, and resulted in a relative EHH (REHH). Then, REHH scores were log-transformed ($\ln (REHH)$) and split into 20 bins with equally-spaced allele frequencies, i.e., with allele frequencies ranging from 0 to 5, 5 to 10 %, and so on. The $\ln (REHH)$ scores were then normalized in each bin to obtain a zero mean and unit variance. The single-SNP scores were further used for analysis of non-overlapping 500-kb windows. To define candidate regions, we used the SNPs that had an LRH greater than 2.6 in each window as test statistic, following a previously reported method [28]. We clustered windows with an increment of 20 SNPs (i.e., windows with less than 20 SNPs were clustered as one group; windows with 20 to 40 SNPs were clustered as one group, and so on) and groups with few windows were excluded. Thus, for both the Holstein and Simmental populations, all windows with less than 40 SNPs or more than 200 SNPs were excluded (see Additional file 1: Figure S1). For each window, we defined the proportion of SNPs with an LRH greater than 2.6 as the *f* value. Within each group, for each window *i*, the *P* value was defined as the fraction of the windows with a higher *f* value than that of window *i*. Windows with *P* values less than 1 % were considered as candidate regions.

### Detection of selection signatures between populations

Highly differentiated genomic regions between these two breeds were detected using two between-population methods, F_ST_ and XP-EHH tests. A Bayesian algorithm proposed by Gianola et al. [29] was used to estimate F_ST_. This method consists of two steps. The first step uses a simple Bayesian model to draw samples from the posterior distribution of *θ*-parameters, i.e., F_ST_. This step assigns a weakly informative prior (${\text{Beta}}\left( {\frac{1}{2}, \frac{1}{2}} \right)$ distribution) to the allele frequencies, and the posterior density of the allelic frequency is ${\text{Beta}}\left( {{\text{n}}_{\text{A}} + \frac{1}{2}, {\text{n}}_{\text{a}} + \frac{1}{2}} \right)$ (${\text{n}}_{\text{A}} , {\text{n}}_{\text{a}}$: the counts of allele A and a in the sample), which is then used to produce the posterior distribution of θ. Let ${\text{p}}_{{{\text{r}},{\text{l}}}}^{{\left( {\text{s}} \right)}}$, ${\text{s}} = 1,2, \ldots ,{\text{S}}$ be a sample from the posterior distribution of ${\text{p}}_{{{\text{r}},{\text{l}}}}$ the frequency of allele ${\text{A}}_{\text{l}}$ at locus ${\text{l}}$ in group ${\text{r}}$, r = 1, 2, …, R. A draw from the posterior distribution of $\uptheta_{\text{l}}$ s given by:

$$\uptheta_{\text{l}}^{{\left( {\text{s}} \right)}} = \frac{{\mathop \sum \nolimits_{{{\text{r}} = 1}}^{\text{R}} \left( {{\text{p}}_{{{\text{r}},{\text{l}}}}^{{\left( {\text{s}} \right)}} } \right)^{2} - \frac{{\left( {\mathop \sum \nolimits_{{{\text{r}} = 1}}^{\text{R}} {\text{p}}_{{{\text{r}},{\text{l}}}}^{{\left( {\text{s}} \right)}} } \right)^{2} }}{\text{R}}}}{{\left( {\frac{{{\text{R}}\mathop \sum \nolimits_{{{\text{r}} = 1}}^{\text{R}} {\text{p}}_{{{\text{r}},{\text{l}}}}^{{\left( {\text{s}} \right)}} - \left( {\mathop \sum \nolimits_{{{\text{r}} = 1}}^{\text{R}} {\text{p}}_{{{\text{r}},{\text{l}}}}^{{\left( {\text{s}} \right)}} } \right)^{2} }}{\text{R}}} \right)}}$$

Then, from S samples, the mean of the posterior distribution of θ_1_ can be calculated and taken as the point estimate of θ_1_. In the second step, the distribution of the θ values across the loci was used to explore the underlying structure, presumably caused by different evolutionary forces, e.g., neutral, balancing or directional selection. By using the FlexMix package [30] in the R project, a sequence of finite mixture models was implemented to fit to θ values, and we were able to observe a mixture of distributions that resulted in clusters representing the different types of acting mechanisms. Model parameters were estimated by maximum likelihood via the expectation–maximization algorithm. The locus was assigned to the component with the largest conditional probability. Models with different numbers of components were compared using Akaike’s information criterion (AIC), and the one with the smallest AIC was preferred. The components with the highest and lowest means of θ values were supposed to be possible signals left by directional and balancing selection, respectively.

To identify highly differentiated regions, we divided the genome into non-overlapping 500-kb windows. The F_ST_ value was calculated for each SNP and then averaged over the SNPs located in each window. The averaged F_ST_ value was used as the test statistic. Windows that were located at the extreme 2.5 % of the empirical distribution were considered as candidate regions for positive selection, as described by Qanbari et al. [9].

Furthermore, to clarify how natural and artificial selection have shaped population differentiation, we employed the approach proposed by Barreiro et al. [31]. SNPs were classified into the following five classes: non-genic, intronic, exonic, 5′ UTR and 3′ UTR. Since demography shapes genome-wide genetic variation, any difference in the degree of differentiation between SNP classes is expected to result from the process of selection, rather than demography. The number of low-F_ST_ SNPs among non-genic SNPs was compared with the number of each of the last four SNP classes using the Chi square test of independence. In this analysis, non-genic SNPs were assumed to be neutral. Therefore, an excess of low-F_ST_ SNPs in one SNP class indicates potential balancing or negative selection, and an excess of high F_ST_ SNPs suggests potential positive selection.

For the XP-EHH test [4], the statistics were calculated in both directions and for all SNPs using the software package coded by Joseph Pickrell [28]. The definition of candidate windows was the same as in the method for the LRH test, except that the maximum |XP-EHH| value was used as the test statistic for each 500-kb non-overlapping window. We clustered windows by the number of SNPs in increments of 20 SNPs, and all windows with less than 80 SNPs or more than 220 SNPs were excluded to remove the groups with few windows (see Additional file 2: Figure S2). Within each group, for each window *i*, the fraction of the windows with a maximum |XP-EHH| value greater than that of window *i* was used as the empirical *P* value for window *i*. Windows with *P* values less than 1 % were considered as candidate regions. According to the sign of the maximum XP-EHH score in each window, candidate windows could be classified into two groups. Since the Holstein and Simmental populations were treated as populations A and B, windows with a positive maximum XP-EHH score were collected in one group, suggesting potential selection in the Holstein population; the other group, suggesting potential selection in the Simmental population, contains the remaining windows.

### Enrichment analyses

Gene contents in the candidate regions were retrieved from the UCSC Table Brower [32]. Before conducting functional annotation, we selected candidate genes within windows that presented selection signals according to the LRH and XP-EHH tests. For the LRH test, each gene overlapping with a candidate window was given a score corresponding to the F_ST_ average of SNPs localized within the boundary positions of the gene extended by 1 kb upstream and downstream; the gene with the maximum score in each window was selected for functional annotation. For the XP-EHH test, we considered only genes within the 100 kb region around the SNP with the maximum |XP-EHH| score, since XP-EHH peaked more narrowly around the candidate variant than other methods, to detect selection signals [6]. Given that a limited number of genes have been annotated in the bovine genome, first we converted the cattle RefSeq mRNA IDs to orthologous human Ensembl gene IDs from the Ensembl Genes 84 Database by BioMart [33]. Gene Ontology (GO) and KEGG pathway analyses were performed in the Holstein and Simmental populations separately using DAVID 6.8 [34]. The candidate genes were classified into categories by cellular component, molecular function, biological process and pathway and were compared to the human genome background supplied by DAVID.

## Results

### Data quality control

In the Holstein population, a total of 574,166 autosomal SNPs with an average distance of 4.37 ± 6.92 kb between adjacent SNPs were retained, and all 96 individuals passed quality control. The Simmental population dataset after quality control comprised 612,148 autosomal SNPs (with an average distance of 4.10 ± 6.08 kb between adjacent SNPs) for 374 of the 447 individuals. The remaining 73 Simmental individuals were removed because of high rates of missing genotypes (i.e., missing rate per individual >0.05). In the next step of relatedness testing, 40 Holstein and 278 Simmental individuals were excluded because of close relationships, which resulted in 56 and 96 unrelated individuals in the Holstein and Simmental populations, respectively.

### Population structure

In the PCA analyses, after merging the Chinese Holstein population and the dataset downloaded from WIDDE, 2252 individuals with 34,533 SNPs remained; after merging the Chinese Simmental population and the dataset downloaded from WIDDE, 2252 individuals with 35,508 SNPs remained. The Chinese Holstein population clustered together with the European Holstein population (see Additional file 3: Figure S3), and the Chinese Simmental population was located close to the European Simmental population (see Additional file 4: Figure S4). Although a few Simmental individuals were weakly admixed with indicine breeds, we assumed that this weak admixture had a limited effect on the detection of selection signatures in this study. The results of breed assignment analyses (see Additional file 5: Table S1) showed that all Chinese Holstein individuals were assigned to the European Holstein population, and all Chinese Simmental individuals were assigned to the Montbéliarde and Abondance populations. Because the world reference dataset in WIDDE only included populations with at least 15 individuals, the Simmental population, which was present in WIDDE but not included in the world reference dataset, was not involved in the breed assignment analyses. As a result, the Chinese Simmental individuals were assigned to the closest populations, i.e., Montbéliarde and Abondance. Therefore, both the PCA and breed assignment analyses confirmed that the Chinese Holstein and Simmental populations originated from Europe.

### Pattern of haplotype blocks

**Fig. 1:**
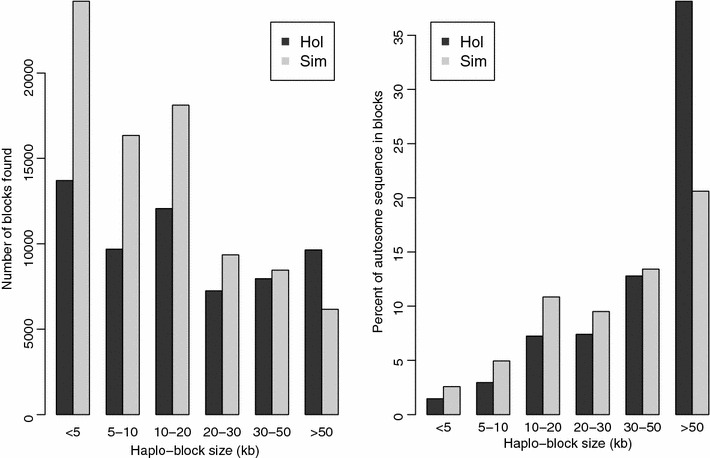
Distribution of haplo-block size in the genomes of Holstein and Simmental cattle

The definition of haplotype blocks for two breeds was determined via Haploview [27]. The distribution of haplotype block sizes is in Fig. 1. In the Holstein population, 504,049 SNPs formed 60,283 blocks that included more than two SNPs for all individuals, with an average of 8.36 SNPs and a mean size of 28.0 ± 38.8 kb per block. In the Simmental population, 514,803 SNPs formed 82,619 blocks that included at least two SNPs for all individuals, with an average of 6.23 SNPs per block. The mean length of the blocks was 18.1 ± 23.7 kb.

### Detection of selection signatures within populations

**Fig. 2:**
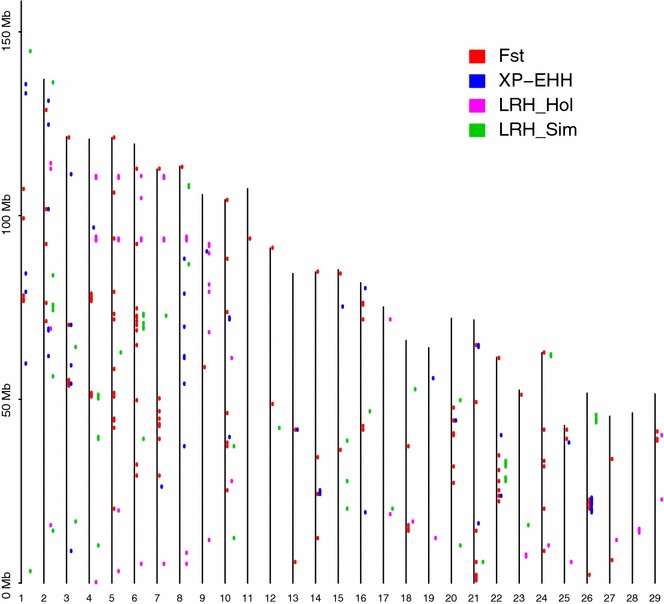
Distribution of selection signatures across the genomes of Holstein and Simmental cattle

A total of 1,325,248 and 2,409,830 LRH tests were performed for the Holstein and Simmental populations. The distributions of the LRH statistics in the two populations are in Figure S5 (see Additional file 6: Figure S5). 0.57 and 0.55 % of the LRH values were higher than 2.6 in the Holstein and Simmental data, respectively. The LRH tests resulted in 54 candidate windows for each population. However, none of them overlapped between the two populations, which suggests that selection may have affected different loci in these two breeds. Figure 2 shows a plot diagram that visualizes the distribution of selection signatures across the genome. Corresponding statistics and genes of interest in the genomic regions that exhibited extreme peaks across breeds are in Table 1. These genes were mainly related to reproduction and production traits, i.e., milk production and growth traits.

**Table 1:** Summary of the most interesting candidate genes within extreme signals

Chr	Position (Mb)	Methods	Candidate gene	Function or full name
1	61.5–62.0	XP-EHH (Hol)	*R3HDM1*	Feed efficiency
4	50.5–51.0	LRH (Sim), F_ST_	*CAV1*, *CAV2*	Lipolytic enzymes
4	77.5–78.0	F_ST_	*AEBP1*	Adipogenesis
4	93.0–93.5	LRH (Hol)	*LEP*	Milk production
5	44.0–45.0	F_ST_	*LYZ* cluster	Immune stress; Rumen digestion
7	5.0–5.5	LRH (Hol)	*MYO3B*	Resistance to bovine tuberculosis
16	42.5–43.0	F_ST_	*AGTRAP*	Mammary gland
18	14.5–15.0	F_ST_	*MC1R*	Coloration
20	31.5–32.0	F_ST_	*GHR*	Milk production
21	5.0–5.5	F_ST_	*CHSY1*	Bone development

### Selection signatures between populations

Overall, F_ST_ values for SNPs ranged from 0.02 to 0.05 with an average of 0.039, which was similar to the results observed by Qanbari et al. [9]. These low F_ST_ indicate the close relationship between these two breeds although they were selected for different breeding goals. The genome-wide distributions of the average F_ST_ and maximum XP-EHH values for each 500-kb window are in Additional file 7: Figure S6. The F_ST_ test identified 127 candidate windows and the XP-EHH test identified 52 candidate windows, with respectively 48 and 4 of them including selection signals in the Holstein and Simmental populations, respectively.

### Overall comparison of selection signals between methods

As expected, each individual test can detect regions that display selection signals according to the features of the genetic polymorphism data. Distributions of overlapping candidate windows among the three methods, i.e., LRH, F_ST_, and XP-EHH, are summarized in Table 2 and Additional file 8: Table S2.

**Table 2:** Summary of overlapping windows with the highest score

	LRH (Sim)	F_ST_	XP-EHH (Hol)	XP-EHH (Sim)
LRH (Hol)	0	1	1	0
LRH (Sim)		6	0	0
F_ST_			12	0

Clearly, only a small proportion of signals overlapped between these tests. For example, the signals obtained by the F_ST_ test and XP-EHH for the Holstein population displayed the largest number of overlapping windows (n = 12), while for the Simmental population there were only six overlapping windows. However, no candidate region was found by all three methods, which is attributed to the different characteristics of these methods and further confirms that the integration of various detection methods should increase the sensitivity of the detection of selection signatures.

### Gene content analyses within genomic regions with selection signals

The annotation of candidate regions that were detected by LRH, XP-EHH and F_ST_ revealed a panel of functionally important genes, such as *growth hormone receptor* (*GHR*), *AE binding protein 1* (*AEBP1*), *chondroitin sulfate synthase 1* (*CHSY1*), *ubiquitin protein ligase E3A* (*UBE3A*) and *myosin IIIB* (*MYO3B*), which are involved in growth, milk production, reproduction and immune response. For example, *GHR* is a well-known gene related to milk production. Several association studies [35–37] have confirmed its major effect on milk yield and composition. The LRH test revealed a selection signal for the *MYO3B* gene in the Holstein population. This gene was previously identified as a quantitative trait locus (QTL) related to resistance to bovine tuberculosis [38].

For enrichment analysis, we selected genes within the candidate windows that were detected by the LRH and XP-EHH tests. Seventy-seven genes (37 genes identified by the LRH test and 40 genes by the XP-EHH test) and 43 genes (38 genes by the LRH test and 5 genes by the XP-EHH test) remained for the Holstein and Simmental data, respectively, as shown in Additional file 9: Table S3. Additional file 10: Table S4 shows the enriched terms based on these two sets of genes. However, none of them reached the level of significance after Benjamini-Hochberg FDR correction [39]. For the Holstein data, enrichments were found for ubiquitin conjugation (ubiquitin-conjugating enzyme activity and ubiquitin-mediated proteolysis), embryo development (embryonic hindlimb morphogenesis) and the immune system (response to lipopolysaccharide) and for the Simmental data, the enriched terms were related to reproduction (spermatid nucleus differentiation, acrosome assembly), protein binding, blood coagulation and metabolic process.

### Impact of selection on population differentiation

**Fig. 3:**
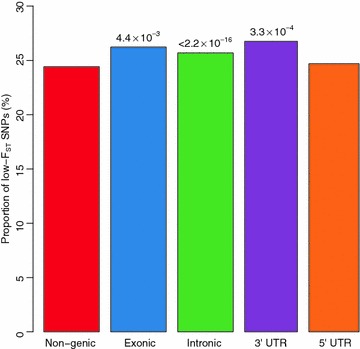
Excess of low-F_ST_ SNPs for different SNP classes with respect to non-genic regions. *P* value is provided when the difference reaches significance level

**Fig. 4:**
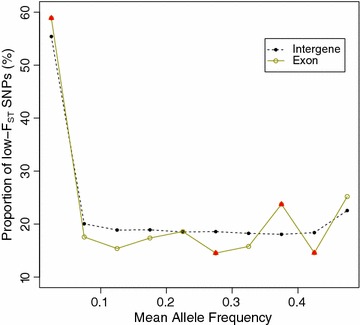
Enrichment of low-F_ST_ SNPs among MAF (minor allele frequency) bins. The *triangle* indicates the difference between exon and non-genic regions that reach the significance level

By comparing the degree of population differentiation among different classes of SNPs (non-genic, intronic, exonic, 5′ UTR, 3′ UTR), we found that the estimated mean F_ST_ values were similar for different classes of SNPs and concordant with the genome-wide estimate, with the highest mean F_ST_ value reaching 0.039 in the 3′ UTR and 5′ UTR regions. Therefore, we investigated the distribution of SNPs with high or low F_ST_ values among different classes. By fitting the distribution of F_ST_ values over loci into a sequence of finite mixture models, SNPs were classified into six clusters (see Additional file 11: Figure S7; Additional file 12: Table S5). Among these clusters, Component 2, including 28,979 SNPs with a high F_ST_, was representative of potential directional selection, whereas the 180,959 SNPs in Component 4 were assumed under potential purifying or balancing selection. Compared to non-genic SNPs, the exon, intron and 3′ UTR classes all presented a significant excess of low-F_ST_ SNPs, which was particularly marked in introns (Fig. 3). To determine why the proportion of low-F_ST_ SNPs was higher in the exons, we split the different SNP classes into 10 equally-sized bins based on the unweighted means of minor allele frequencies in these two populations. As shown in Fig. 4, in the low-MAF bin (MAF ranging from 0 to 0.05), the proportion of low-F_ST_ SNPs was significantly higher in exons than in non-genic regions. This result is consistent with the stronger purifying selection on exons.

## Discussion

In this study, we implemented three tests based on allele frequency and LD to detect the genome-wide footprints left by artificial selection in Holstein and Simmental populations. Our results revealed a series of well-known and novel genes, such as the *GHR* and *LEP* (*leptin*) genes that are related to milk production, *MC1R* (*melanocortin 1 receptor*), which is involved in coat color, and *MYO3B* and *FCRL4* (*Fc receptor like 4*), which are involved in the regulation of immune response. We also provided insight into the process of population differentiation between the Holstein and Simmental breeds and identified divergent artificial selection and strong purifying selection. Distinguishing recent positive selection from the confounding effect of population demographic history continues to be a major challenge. Studies using new and powerful tests are needed to confirm and refine our results.

In this study, we focused mainly on detecting the footprints of artificial selection left in the Holstein and Simmental populations after the process of domestication. These two breeds were selected because they had distinct phenotypes but close genetic relationships. On the one hand, Holstein, as a dairy breed, is distinguished for its high milk production, whereas Simmental is a dual-purpose breed. The different breeding goals are expected to intensify the differentiation between these two breeds. On the other hand, the F_ST_ value between these two breeds was reported to be very low (F_ST_ = 0.04) by Qanbari et al. [9], which indicates a very close relationship. Our results confirm these previous findings. One explanation for the unexpectedly close relationship is that artificial selection is the primary cause of the distinct phenotypic traits between Holstein and Simmental cattle. The genome-wide scan for selection signatures by comparing relatively close populations, such as Holstein and Simmental, is unlikely to be confounded by demographic history and ascertainment bias.

Compared with previous studies using the Bovine 50K SNP chip, the high-density SNP panel is expected to offer additional accuracy. Since false positives may occur at any one site by chance, regions with a set of positive SNPs are more likely to be true signals [40]. Thus, window-based approaches incorporating signals across multiple sites can improve the power and reduce the rate of false positives. However, in the Bovine 50K SNP chip, there are only approximately six SNPs per 500-kb window [9], which is unlikely to efficiently reduce false discovery rate, whereas the average size of windows that contain more than 110 SNPs should be reasonably large in our study. In addition, for haplotype-based tests, the high-density SNP panel is expected to provide more reliable and comprehensive LD information. Furthermore, the low-density SNP panel is very poor for implementing the LRH test based on haplotype blocks defined by Gabriel et al. [26], which is widely used for the detection of selection signatures [17, 19, 41]. The low SNP density may lead to two biases in the detection of haplotype blocks, as noted by Gabriel et al. [26]. First, small blocks are less likely to be discovered in regions with few SNPs. Conversely, identified blocks tend to extend beyond the boundaries of the blocks. Thus, Villa-Angulo et al. [42] suggested that at least 574,000 SNPs would be necessary to characterize the haplotype block structure across the entire genome. As shown in Fig. 1, although most of the SNPs were located within large blocks in both Holsteins and Simmentals, the majority of the blocks were less than 50 kb. We found a mean block size of approximately 20 kb, which is less than the average distance between adjacent markers in the Illumina Bovine 54K SNP chip. Accordingly, most blocks would be missed or enlarged in low-density SNP panels, which would weaken the power of the LRH test using core haplotype blocks.

In comparison to the human haplotype map [1], the average block size observed in this study is slightly larger than that observed within the human ENCODE regions which range from 7.3 kb in YRI (Yoruba from Ibadan, Nigeria) to 16.3 kb in CEU (Utah residents with ancestry from northern and western Europe). This difference can be partially attributed to the smaller effective population size of cattle, which is due to the significant population bottlenecks that occurred during the process of domestication and the establishment of modern breeds [43]. However, it must be noted that this difference could also be due to other factors, such as SNP density, sample size and population structure.

To detect selection signals, we implemented three complementary methods, i.e. LRH, XP-EHH and F_ST_, which each have their own features. Both the F_ST_ and XP-EHH tests are based on population differentiation, but they are complementary in time scale. The F_ST_ test compares the variation of allele frequencies within and between populations, and the locus that shows the largest differences in allele frequencies between populations is assumed to be a signal of selection. The XP-EHH test compares the extended haplotype homozygosity between populations. Compared to allele frequency difference, long-range haplotypes persist for relatively short periods of time before being broken down by recombination [44]. Thus, the F_ST_ test is more efficient for detecting ancient positive selection than the XP-EHH test. Moreover, as mentioned above, F_ST_ exploits the different patterns of polymorphism between recently diverged populations and thus is powerful for detecting selection on standing variation [14]. Both LRH and XP-EHH tests are designed to detect recent selection signatures, whereas LRH has more power to detect incomplete sweeps, and XP-EHH is efficient for detecting selected alleles that are near or at fixation [4]. Since the domestication of cattle occurred ~10,000 years ago, these three methods are expected to detect the signals of selection during different periods of cattle domestication and breeding. Notably, although there is a difference in sample size between the two populations, its impact on these three methods is expected to be small. For the F_ST_ test, the posterior density of the allelic frequency is generated by ${\text{Beta}}\left( {{\text{n}}_{\text{A}} + \frac{1}{2}, {\text{n}}_{\text{a}} + \frac{1}{2}} \right)$ to account for the sample size effect [29]. For the LRH and XP-EHH tests, few samples are required to maintain the power of iHS and XP-EHH [28]. Accounting for the similar power of LRH and iHS, the difference in sample size is not expected to influence markedly the power of LRH.

We identified 102 and 58 windows that displayed evidence of selection for the Holstein and Simmental populations, respectively. There were no overlapping windows between these two populations. The follow-up enrichment analyses also resulted in terms that are related to different biological functions, although none of them reached the level of significance after Benjamini–Hochberg FDR correction [39]. For example, terms associated with proteolysis, embryo development and immune response were found for the Holstein population and terms involved in reproduction and metabolism were found for the Simmental population. Our findings suggest that these two populations may have been divergently selected for different loci.

Comparing the overlapping windows that were detected between methods (see Additional file 8: Table S2) with the core selective sweep (CSS) regions reported in [45], we found a list of common candidate windows and interesting genes. For example, in our study the F_ST_ and LRH tests on the Holstein population suggested that selection occurred in the window between 93.5 and 94 Mb on chromosome 5. This window is located within CSS-113 that spans the region between 91.8 and 94.4 Mb and was identified in three breeds [45]. This window was annotated as harboring the *MGST1* (*microsomal glutathione S*-*transferase 1*) gene, which was identified as a candidate gene for milk composition (including fat yield and percentage) in a multibreed genome-wide association study [46]. Two sequence-based association tests [47, 48] further confirmed the association between *MGST1* variants and milk composition. Moreover, eQTL mapping with a high-depth mammary RNA sequence dataset revealed that the expression of MGST1was associated with a QTL genotype for fat percentage [48]. Therefore, taken together, these studies confirm *MGST1* as the causal gene affecting milk composition. Another window (between 50.5 and 51 Mb on chromosome 4), in which signals of selection were detected by the F_ST_ and LRH tests for the Simmental population, contained CSS-88 that is part of the only selection signature identified in Fleckvieh cattle [49], which is a dual-purpose breed like the Simmental breed. This window encompasses the *CAV1* and *CAV2* (*caveolin 1* and *2*) genes, which play an important role in the control of lipolysis [50, 51].

**Fig. 5:**
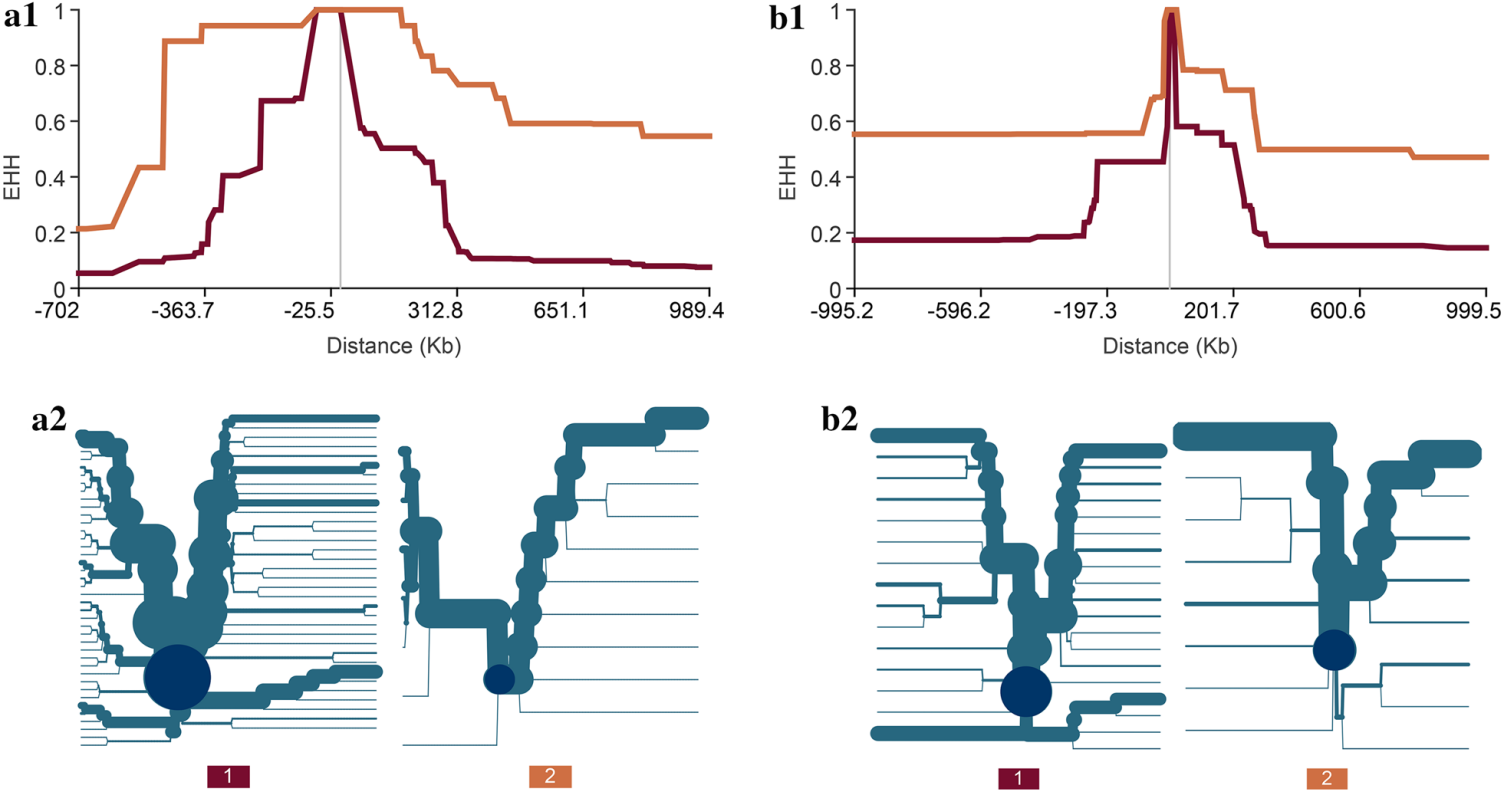
EHH versus distance charts and haplotype bifurcation diagrams for *DGAT1* (**a**) and *GHR* (**b**). EHH versus distance charts (1) and haplotype bifurcation diagrams (2) were plotted with Sweep 1.1. The *closest markers* (ARS-BFGL-NGS-4939 for *DGAT1* and BovineHD2000009188 for *GHR*) to causal mutations were used as core SNPs. The haplotype bifurcation diagram is bi-directional with the root representing a core SNP. The *thickness of the lines* corresponds to the frequency of the indicated haplotype

It is worth noting that the LRH test failed to detect strong signals of selection in the regions that contain genes that are known to be related to milk production, such as *DGAT1* and *GHR*, in the Holstein population. Thus, we examined the windows that include these two genes, i.e., window 1.5–2.0 Mb on BTA14 for the *DGAT1* gene and windows 31.5–32.0 and 32.0–32.5 Mb on BTA20 which are crossed by the *GHR* gene. We found 40, 109 and 78 SNPs in these three windows, respectively, but no EHH or REHH value was calculated from these SNPs. We hypothesize that this might be due to the extensive LD in the vicinity of the *DGAT1* and *GHR* genes (see Additional file 13: Figure S8), which prevents the LRH test to identify any SNP with an EHH between 0.03 and 0.05 in a 1-Mb area from the core SNP. For these two genes, we chose the closest SNP near the causal mutation, and calculated its REHH at a 1-Mb distance instead of the marker H 0.04. As shown in Fig. 5, each of these genes had one allele that displayed more extended haplotype homozygosity than the other, which is a clear signal of recent selection. This analysis showed that the extensive LD present in the cattle genome may result in our single-SNP LRH test missing some recent selection signatures.

Finally, in the analysis of the impact of human-mediated selection on population differentiation, we found that low-F_ST_ SNPs were enriched in the low-MAF bin (MAF: 0–0.05) in exons compared to non-genic regions. The enrichment is most likely caused by purifying selection on exons. Moreover, there was little difference in MAF distributions between non-genic regions and exons (see Additional file 14: Figure S9), which excluded the possibility that MAF distribution within the low-MAF bin might influence the proportion of low-F_ST_ SNPs.

## Conclusions

Using a complementary analysis based on the 770K high-density SNP chip, we constructed a high-resolution map of selection signatures for Chinese cattle populations, which increases the spectrum of selective signals in the cattle genome. In addition, the selective signals identified in this study clearly reflected the stronger purifying selection that occurred in the exons of the bovine genome. This result provides further insight into the genome evolution and selection mechanisms in cattle.

## Additional files

10.1186/s12711-016-0254-5 Number of SNPs in 500-kb windows for the LRH test.

10.1186/s12711-016-0254-5 Number of SNPs in 500-kb windows for the XP-EHH test.

10.1186/s12711-016-0254-5 PCA analysis on Chinese Holstein population (HOL_CHI) with the world reference dataset and the Simmental reference population included in WIDDE.

10.1186/s12711-016-0254-5 PCA analysis on Chinese Simmental population (SIM_CHI) with the world reference dataset and the Simmental reference population included in WIDDE.

10.1186/s12711-016-0254-5 The top 5 genetically closest populations to Chinese Holstein and Simmental individuals in breed assignment analyses.

10.1186/s12711-016-0254-5 Genome-wide distribution of SNP-based LRH values for Holstein and Simmental. The dash line indicates the threshold for the LRH test (LRH > 2.6).

10.1186/s12711-016-0254-5 Genome-wide distribution of 500-kb window-based maximum |XP-EHH| and average F_ST_. The dash line indicates the threshold for the F_ST_ test.

10.1186/s12711-016-0254-5 Overlapping windows between LRH, XP-EHH and F_ST_ tests.

10.1186/s12711-016-0254-5 Candidate genes used for functional annotation.

10.1186/s12711-016-0254-5 DAVID analyses on candidate genes.

10.1186/s12711-016-0254-5 Rootgrams of the posterior class probability for F_ST_ values.

10.1186/s12711-016-0254-5 Distribution of six components inferred by FlexMix analysis and number of SNPs in each component.

10.1186/s12711-016-0254-5 A graphical representation of pairwise D’ for the *DGAT1* region (A) and *GHR* region (B).

10.1186/s12711-016-0254-5 Distribution of unweighted means of minor allele frequencies for non-genic and exonic SNPs in the low-MAF bin (0-0.05).
