# Supplementary figures and images for: Identification of selective sweeps reveals divergent selection between Chinese Holstein and Simmental cattle populations
Source: Genet Sel Evol. 2016 Oct 6;48:76. doi: 10.1186/s12711-016-0254-5 (PMC5054554; doi:10.1186/s12711-016-0254-5)

**Holstein**

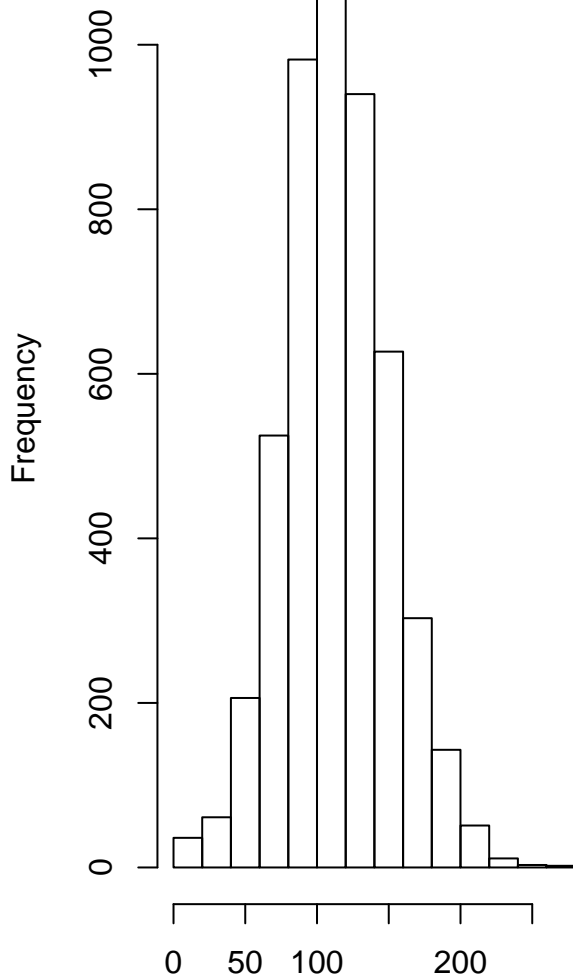

Number of SNPs in 500 kb windows

**Simmental**

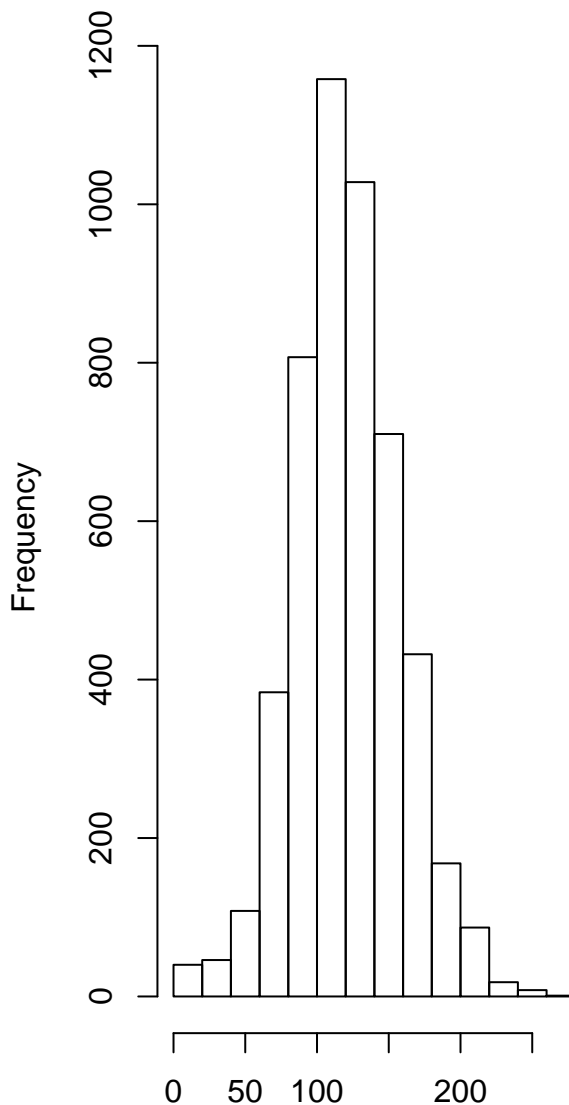

Number of SNPs in 500 kb windows

Supplement: Supplementary file 1 — 10.1186/s12711-016-0254-5 Number of SNPs in 500-kb windows for the LRH test. [file 12711_2016_254_MOESM1_ESM.pdf]

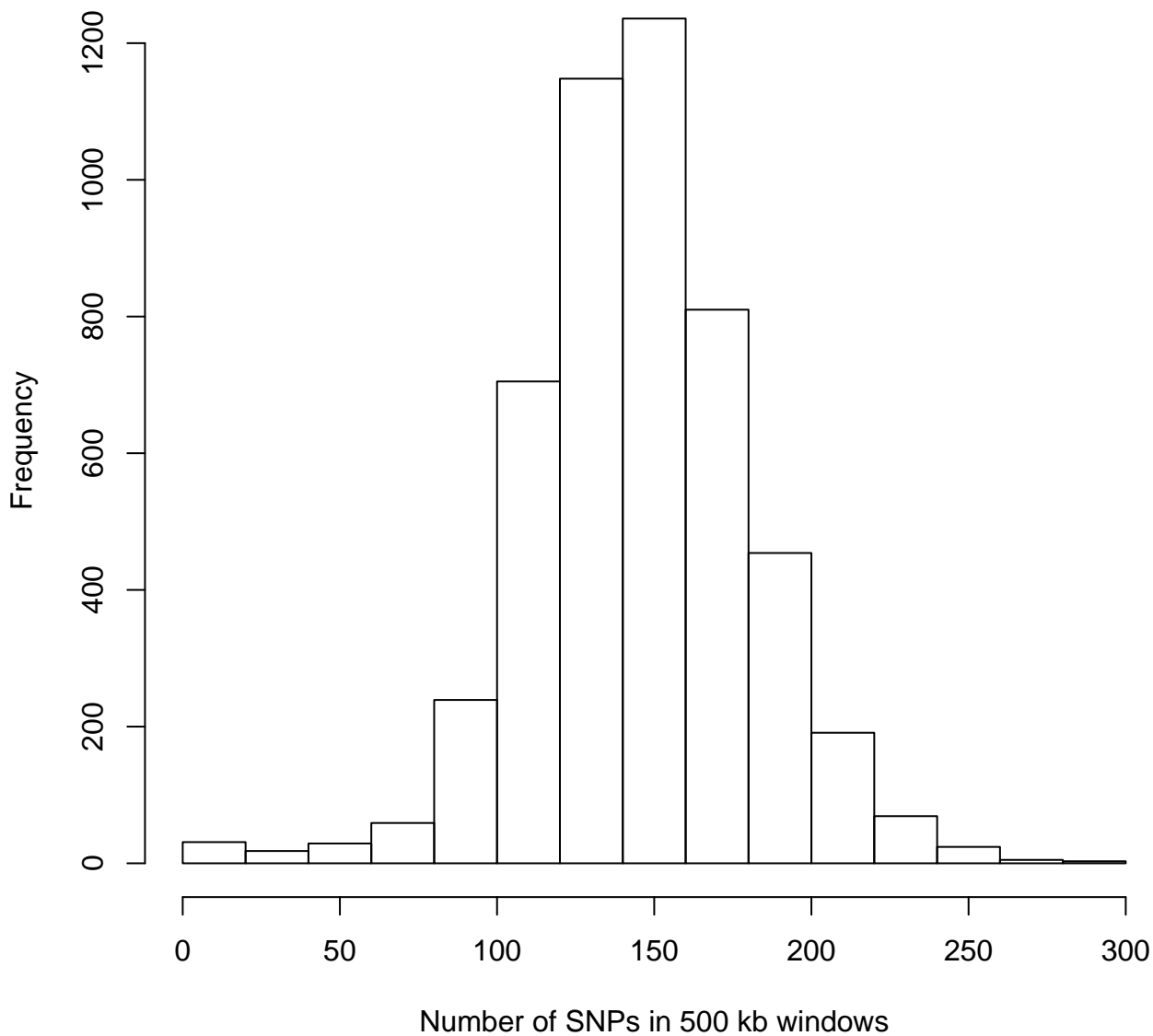

Supplement: Supplementary file 2 — 10.1186/s12711-016-0254-5 Number of SNPs in 500-kb windows for the XP-EHH test. [file 12711_2016_254_MOESM2_ESM.pdf]

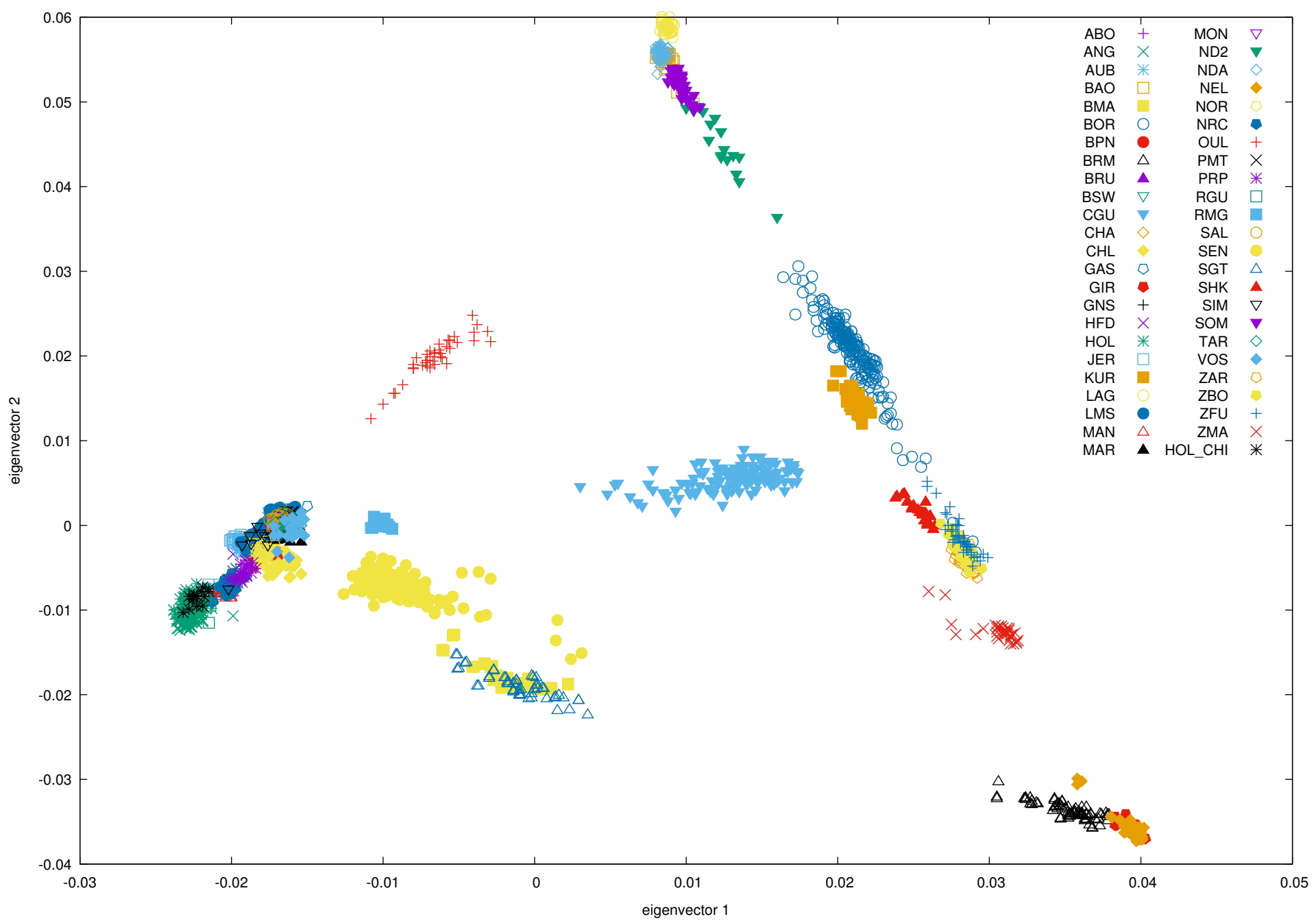

Supplement: Supplementary file 3 — 10.1186/s12711-016-0254-5 PCA analysis on Chinese Holstein population (HOL_CHI) with the world reference dataset and the Simmental reference population included in WIDDE. [file 12711_2016_254_MOESM3_ESM.pdf]

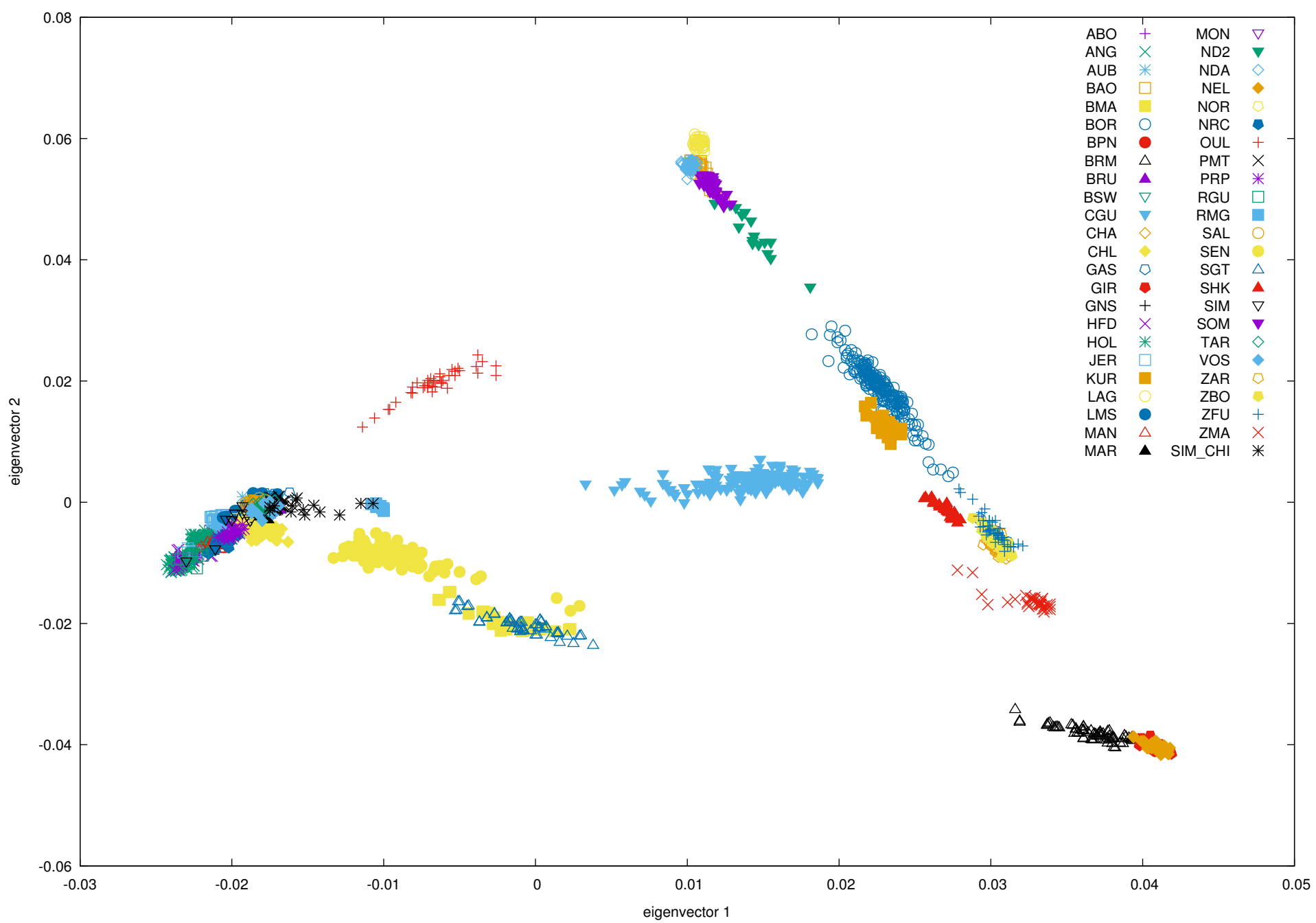

Supplement: Supplementary file 4 — 10.1186/s12711-016-0254-5 PCA analysis on Chinese Simmental population (SIM_CHI) with the world reference dataset and the Simmental reference population included in WIDDE. [file 12711_2016_254_MOESM4_ESM.pdf]

**Holstein**

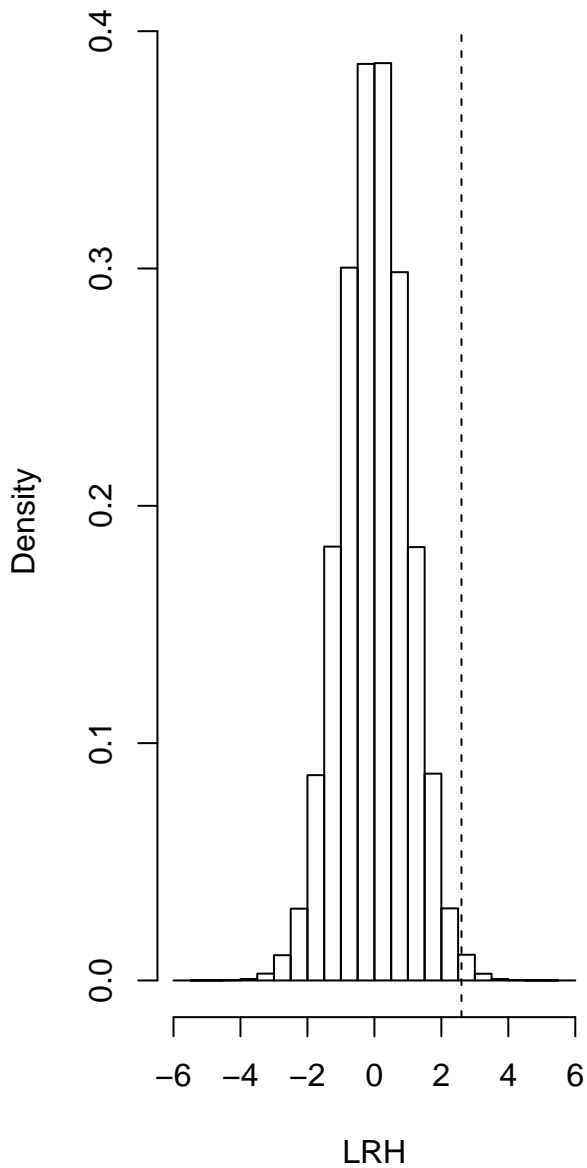

**Simmental**

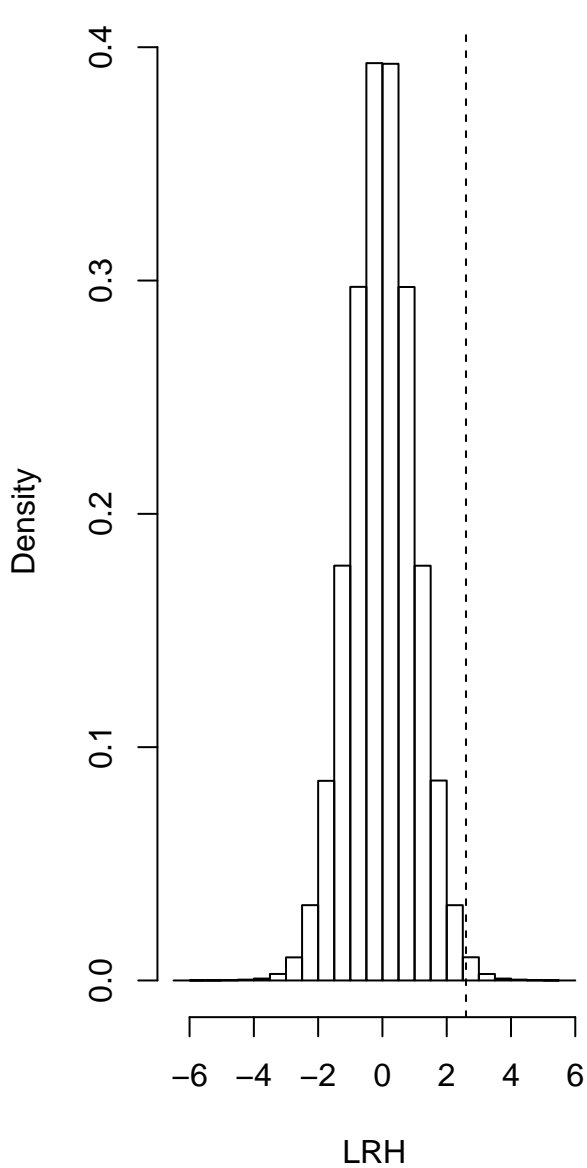

Supplement: Supplementary file 6 — 10.1186/s12711-016-0254-5 Genome-wide distribution of SNP-based LRH values for Holstein and Simmental. The dash line indicates the threshold for the LRH test (LRH > 2.6). [file 12711_2016_254_MOESM6_ESM.pdf]

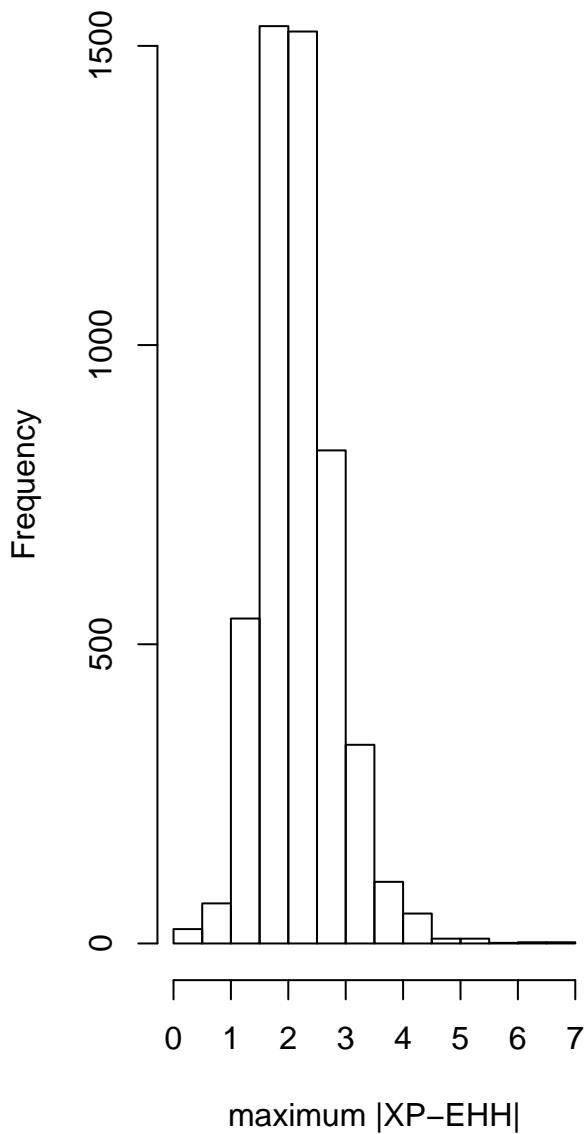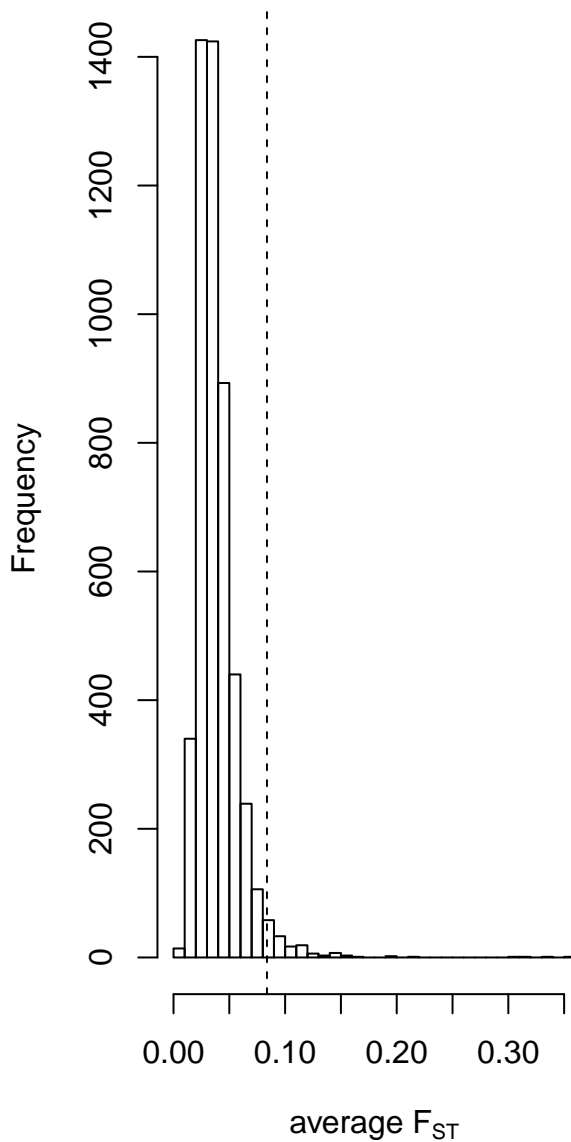

Supplement: Supplementary file 7 — 10.1186/s12711-016-0254-5 Genome-wide distribution of 500-kb window-based maximum |XP-EHH| and average FST. The dash line indicates the threshold for the FST test. [file 12711_2016_254_MOESM7_ESM.pdf]

# Rootogram of posterior probabilities > 1e-04

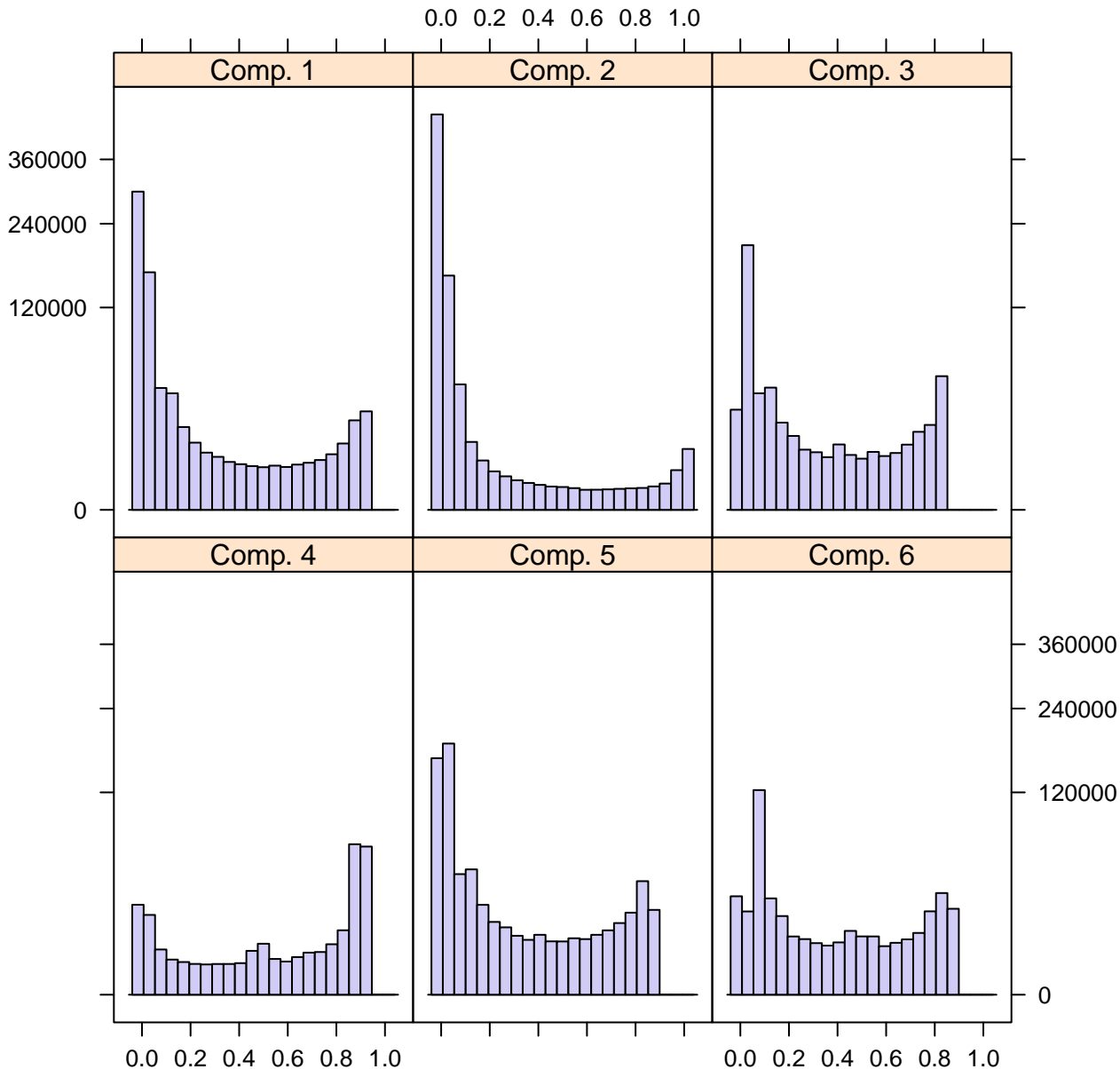

Supplement: Supplementary file 11 — 10.1186/s12711-016-0254-5 Rootgrams of the posterior class probability for FST values. [file 12711_2016_254_MOESM11_ESM.pdf]

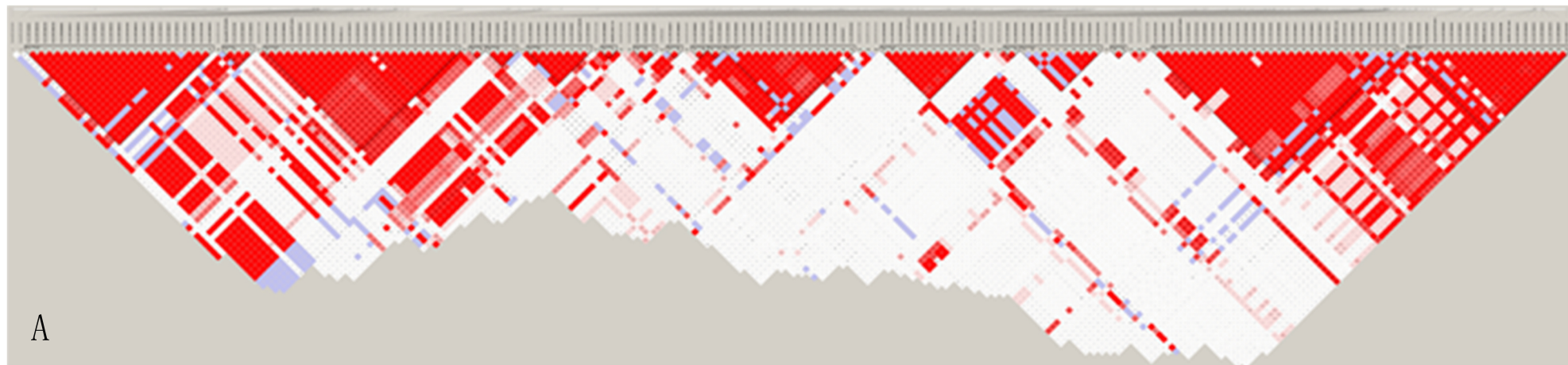

A

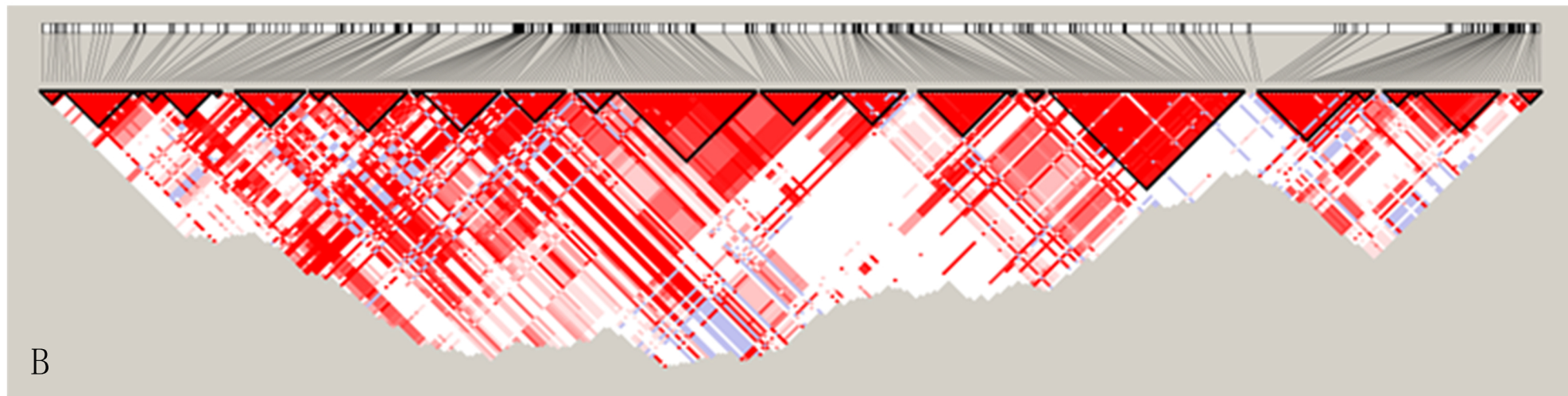

B

Supplement: Supplementary file 13 — 10.1186/s12711-016-0254-5 A graphical representation of pairwise D’ for the DGAT1 region (A) and GHR region (B). [file 12711_2016_254_MOESM13_ESM.pdf]

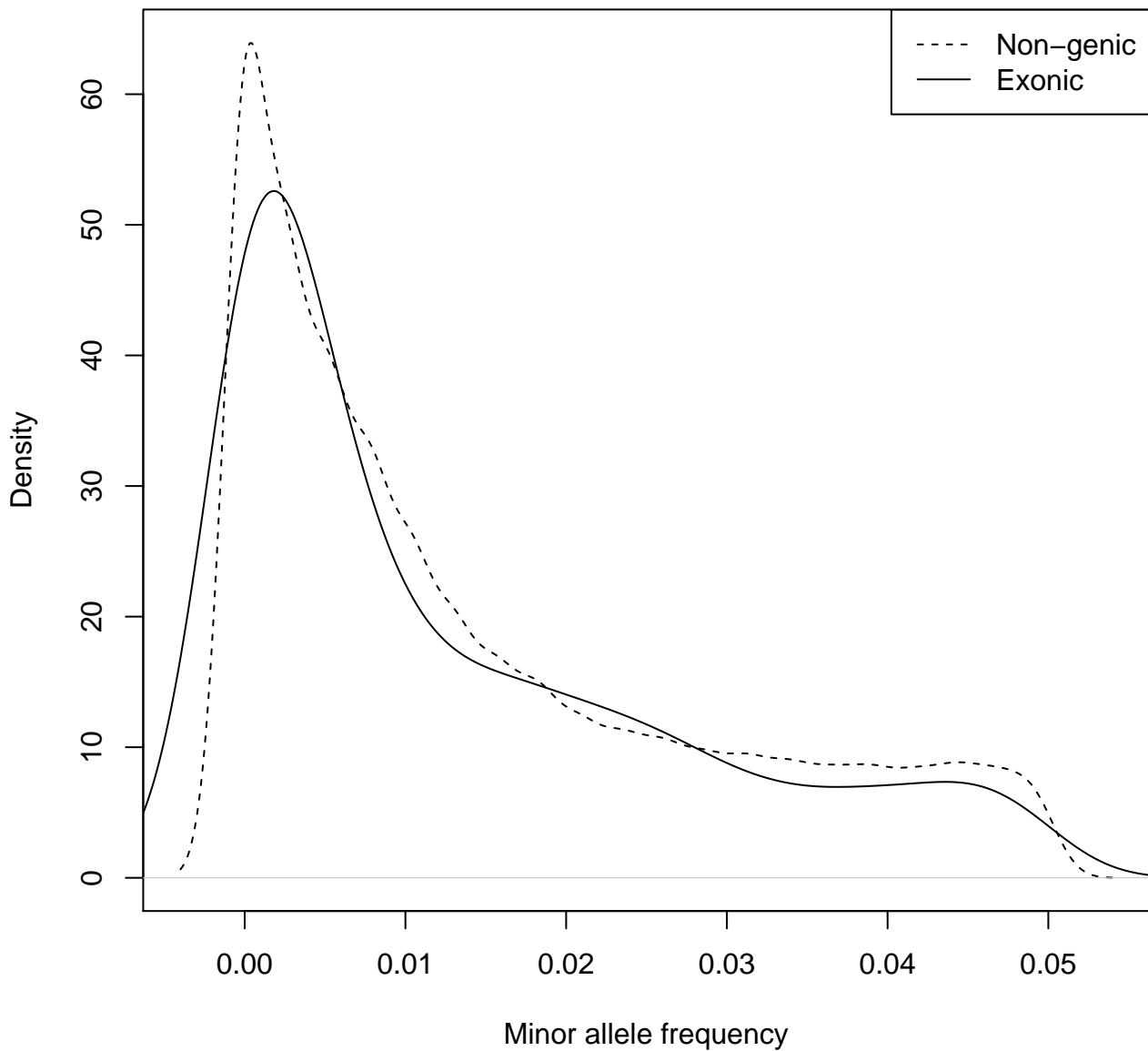

Supplement: Supplementary file 14 — 10.1186/s12711-016-0254-5 Distribution of unweighted means of minor allele frequencies for non-genic and exonic SNPs in the low-MAF bin (0-0.05). [file 12711_2016_254_MOESM14_ESM.pdf]
